# Validation of Candidate Gene-Based Markers and Identification of Novel Loci for Thousand-Grain Weight in Spring Bread Wheat

**DOI:** 10.3389/fpls.2019.01189

**Published:** 2019-09-26

**Authors:** Deepmala Sehgal, Suchismita Mondal, Carlos Guzman, Guillermo Garcia Barrios, Carolina Franco, Ravi Singh, Susanne Dreisigacker

**Affiliations:** ^1^Department of Bioscience, CIMMYT, Texcoco, Mexico; ^2^Departamento de Genética, Escuela Técnica Superior de Ingeniería Agronómica y de Montes, Edificio Gregor Mendel, Campus de Rabanales, Universidad de Córdoba, Córdoba, Spain; ^3^Recursos Genéticos y Productividad, Colegio de Postgraduados, Montecillo, Mexico

**Keywords:** *Triticum aestivum*, thousand-grain weight, genotyping-by-sequencing, haplotypes, elite yield trial

## Abstract

Increased thousand-grain weight (TGW) is an important breeding target for indirectly improving grain yield (GY). Fourteen reported candidate genes known to enhance TGW were evaluated in two independent and existing datasets of wheat at CIMMYT, the Elite Yield Trial (EYT) from 2015 to 2016 (EYT2015-16) and the Wheat Association Mapping Initiative (WAMI) panel, to study their allele effects on TGW and to verify their suitability for marker-assisted selection. Of these, significant associations were detected for only one gene (*TaGs3-D1*) in the EYT2015-16 and two genes (*TaTGW6* and *TaSus1*) in WAMI. The reported favorable alleles of *TaGs3-D1* and *TaTGW6* genes decreased TGW in the datasets. A haplotype-based genome wide association study was implemented to identify the genetic determinants of TGW on a large set of CIMMYT germplasm (4,302 lines comprising five EYTs), which identified 15 haplotype blocks to be significantly associated with TGW. Four of them, identified on chromosomes 4A, 6A, and 7A, were associated with TGW in at least three EYTs. The locus on chromosome 6A (Hap-6A-13) had the largest effect on TGW and additionally GY with increases of up to 2.60 g and 258 kg/ha, respectively. Discovery of novel TGW loci described in our study expands the opportunities for developing diagnostic markers and for multi-gene pyramiding to derive new allele combinations for enhanced TGW and GY in CIMMYT wheat.

## Introduction

Of all yield components, grain weight is the most stable and heritable trait, hence an important selection target for the genetic improvement of grain yield (GY) (Cooper et al., 2012; Xiao et al., 2012). In wheat, a number of genes related to increased thousand grain weight (TGW) have been cloned in the past decade taking advantage of the synteny between wheat and rice (Jiang et al., 2011; Su et al., 2011; Ma et al., 2012; Yang et al., 2012; Chang et al., 2013; Guo et al., 2013; Dong et al., 2014; Qin et al., 2014; Zhang et al., 2014; Jiang et al., 2015; Hanif et al., 2016; Hu et al., 2016; Ma et al., 2016; Simmonds et al., 2016;Wang et al., 2016; Zhang et al., 2012; Zhang et al., 2015; Zhang et al., 2017; Zheng et al., 2014). This has enriched the understanding of this trait and concomitantly gene-based single nucleotide polymorphism (SNP) markers have become available that are suggested to be used to enhance TGW through marker-assisted selection (MAS). Scrutiny of the published literature, however, has revealed that the effects of most of these genes have not been validated in diverse sets of wheat germplasm.

Recently, Mohler et al. (2016) investigated associations of 13 candidate genes (CGs) with TGW and kernel size traits in a European winter wheat panel. Favorable alleles of nine CGs were present in very low (<5%) or very high (>95%) frequencies in the panel and hence were unsuitable for analysis by CG association approach. Only *TaCwi-A1* showed association with TGW; the reported favorable allele *TaCwi-A1a* (Ma et al., 2012) was associated with increased TGW but decreased GY thus pointing to compensatory effects of the *TaCwi-A1* gene on TGW and GY (Mohler et al., 2016). Similarly, varying effects of observed haplotypes for the gene *TaGW2* have been reported. Su et al. (2011) and Han et al. (2011)suggested the haplotype Hap-6A-A to be favorable for selection of genotypes with higher grain width and TGW, while Zhang et al. (2013) reported the haplotype Hap-6A-G as the favorable allele. Inconclusive results were also obtained in a recent study on CIMMYT germplasm, where Hap-6A-G was found superior in Mexico and the second allele, i.e., Hap-6A-A, in Nepal and India (Sukumaran et al., 2018). These examples suggest that besides compensatory effects on GY, the genetic effects of the reported CGs can be environment- and/or germplasm-dependent. Hence, validation of their effects in diverse germplasm is a prerequisite for their effective deployment in MAS. To accomplish this, we investigated allelic effects of 14 CGs on TGW in two independent and existing panels at CIMMYT; both panels represent CIMMYT wheat germplasm and have different compositions and diversities. One of the panels is an elite yield trial (EYT) composed of spring wheat lines that formed the entries of multi-environment trials during the 2015–2016 growing season. The other panel is the well-known WAMI (Wheat Association Mapping Initiative) that includes historical cultivars and synthetic-derived lines (Lopes et al., 2012; Lopes et al., 2015).

The specific objectives of this study were 1) to test the frequencies of alleles of 14 CGs in two independent panels of wheat, 2) to explore the allele effect of each CG on TGW and GY by implementing CG-based association study, and 3) to identify additional genetic determinants of TGW using a haplotype-based genome wide association study (GWAS) in five recent CIMMYT EYTs.

## Materials and Methods

### Plant Materials and Phenotyping

For CG-based association study, two bread wheat datasets, EYT2015-16 and the WAMI, were used. The EYT2015-16 dataset comprised 829 spring bread wheat breeding lines, which formed the entries of multi-environment trials during the 2015–2016 growing season. The WAMI panel is genetically a more diverse collection and comprised 294 lines with a reduced range of variation in phenology and height (for details on WAMI, refer to Lopes et al., 2012). For haplotype-based GWAS, 4,302 lines that formed the EYT2011-12, EYT2012-13, EYT2013-14, EYT2014-15, and EYT2015-16 and comprised 643, 905, 983, 942, and 829 lines, respectively, were used (**Supplementary Table S1**).

All EYTs were phenotyped at the Norman E. Borlaug Experimental Research Station (CENEB) in Ciudad Obregon, Mexico, during the respective growing seasons. For each EYT, five environments, including optimum and stress environments, were induced at the same site by modulating planting date, field management, and irrigation. The experimental design was an alpha lattice design with three replications. Each experiment included 28 lines and 2 checks. Small size units (30 plots with 28 entries and 2 checks) are used to minimize the field variation, which simplifies selection. TGW was measured in one of the five environments, optimally irrigated as single replications using a Seed Counter Contador (http://www.pfeuffer.com/contador.html?&L=5). Unbroken seeds were put in the Seed Counter and 1,000 seeds were counted by the counter and weighed to reveal TGW in grams. The counted seeds were put in an oven for 48 h at 72°C and TGW was reweighed to get moisture estimates. Adjusted means were calculated as described in Sehgal et al. (2017).

The WAMI population with 294 lines was evaluated for 3 years (2010, 2011, and 2013) under optimal management at CENEB. The trials were timely sown with full irrigation applied through gravity fled-irrigation. The experimental design was an alpha lattice with two replications. Blocks were arranged based on the heading date of the materials. TGW on two replications was measured as described in the manual “Physiological breeding II: a field guide to wheat phenotyping” (Pask et al., 2012). In brief, a random sample of whole grains was cleaned carefully to remove all broken and aborted grains before putting in counting machine. Two hundred grains were counted, re-dried, and weighed (DW_200_G). TGW was then calculated by multiplying DW_200_G by five. In each case, two replicates were measured but if the values of the two replicates differ by more than 10%, then a third sample was taken. Best Unbiased Linear Predictors (BLUPs) were calculated using META-R (Vargas et al., 2013) and were used for analysis.

### Gene-Based and Genome-Wide Genotyping

For the study, we selected 14 CGs that have a direct influence on TGW rather than on grain morphometric traits. Genotyping with the selected CGs was performed using published Kompetitive allele-specific PCR (KASP) assays detailed in CerealsDB (http://www.cerealsdb.uk.net/cerealgenomics/CerealsDB/kasp_download.php?URL=) and in Rasheed et al. (2016). KASP assays for genes *TaCwi-A1* (2A) and *TaGW2* (6A) were designed in-house. **Table S2** enlists each of the 14 CGs and the corresponding SNP markers, and **Table S3** enlists the details of KASP primers designed in-house. For whole genome genotyping, genotyping-by-sequencing (GBS) was applied as described in Rutkoski et al. (2016). Briefly, the genotypes were sequenced at 192-plexing on Illumina HiSeq2000 with 1 × 100 bp reads. SNPs were called across all lines using the TASSEL GBS pipeline as described in Glaubitz et al. (2014) and anchored to the genome assembly of Chinese Spring (International Wheat Genome Sequencing Consortium, 2018).

### Haplotype Block Construction

GBS data of all 4,302 lines were considered together to construct haplotype blocks. Markers with more than 40% missing data were excluded from haplotype construction. Since the haplotype blocks were created on all lines together, a MAF ≥ 0.15 was applied so that in each nursery a MAF ≥0.05 could be achieved. From an initial set of 20,794 SNP markers obtained on the 4,302 samples, a set of 8,443 filtered SNPs was used for haplotype block construction. The blocks were generated based on the linkage disequilibrium (LD) parameter D’ using the R script, but modified as described in Gabriel et al. (2002). We calculated D’ 95% confidence intervals between SNPs and each comparison was categorized as “strong LD,” “inconclusive,” or “strong recombination.” A haplotype block was created if 95% of the comparisons in one block were in “strong LD.” For two or more SNPs to be classified in “strong LD,” the minimum lower and upper confidence interval values were set to 0.70 and 0.98, respectively. The haplotype blocks were named as combinations of the prefix “Hap” for the haplotype block followed by chromosome, and then a number, which represents incrementing number of the haplotype blocks along the chromosome.

### CG and Haplotype-Based GWAS

Population structure was investigated in the five EYTs by principal component analysis (PCA) using the function PRCOMP from the STATS package in R. For the WAMI panel, reported subpopulation structure from Lopes et al. (2015), based on the 1B.1R translocation, was utilized for the analysis. The kinship matrix was calculated by the VanRaden algorithm (VanRaden, 2008) in the GAPIT package (Lipka et al., 2012). For association analysis, a mixed linear model (MLM) was applied with PCA as a fixed variate and kinship as random. Bayesian information criterion (BIC; Schwarz, 1978) was used to assess the appropriate number of principal components for analysis, i.e., PC with the largest BIC value was used as a fixed variate. Candidate gene-based association mapping was conducted in TASSEL v4.0 and haplotype-based GWAS was conducted in Plink v1.07 (Purcell et al., 2007). The allelic effect of the CGs and associated haplotypes was estimated as the difference between the mean value of lines with and without favorable allele and was presented as box plots.

### Epistatic Interactions, Stepwise Regression and *In Silico* Analysis

Two- and three-locus interactions were studied using an in-house script executed in R as described in Sehgal et al. (2017). Forward stepwise regression was conducted using the LEAPS package in R. *In silico* analysis of the significant loci was conducted in Ensembl Plants software and database system (https://plants.ensembl.org/Triticum_aestivum/Info/Index).

## Results

### Phenotypic Variation, CG Allele Frequencies and Association With TGW in EYT2015-16 and WAMI Datasets

Figure S1 shows the distribution of TGW in the EYT and WAMI panels. TGW ranged from 33.5 to 57.8 g, 39.2 to 61.3 g, 36.2 to 57.0 g, 34.5 to 57.6 g, and 34.9 to 55.5 g in EYT2011-12, EYT2012-13, EYT2013-14, EYT2014-15, and EYT2015-16, respectively. In the WAMI panel, TGW was, in general, lower than in the EYTs and ranged from 33.8 to 54.7 g, 32.2 to 54.8 g, and 31.7 to 52.8 g in year 2010, 2011, and 2013, respectively. Pearson’s correlations of TGW with GY varied across EYTs (years) and induced environments between negative values (-0.18, *P* < 0.000) and positive values (0.39, *P* < 0.000) (**Supplementary Table S4**).

The frequencies of the 14 CGs in the two wheat datasets are shown in **Table 1**. The favorable alleles of the three genes, *TaSus2-2B, TaCKX6-D1*, and *TaGASR7-A1*, were absent or had very low frequencies (<5%) in both datasets. The favorable allele of the gene *TaCwi-A1* had low frequency in EYT2015-16 (0.6%) while that of *TaTPP-6AL* gene was present in 96.5% of lines. The favorable alleles of the remaining 10 genes were present in moderate to high frequencies in EYT2015-16 (19.5% to 85.2%). In WAMI, the favorable allele of the gene *TaCwi-5D* was present in 99% of lines while those of the remaining genes were in low (6.4%) to high (87.7%) frequencies. Since both alleles of the genes *TaGW2-6A* and *TaSus1-7A* are reported to be favorable, we considered both allele frequencies in our datasets. The haplotype Hap-A-A of *TaGW2-6A* was moderately frequent (30% to 32%), whereas Hap-A-G was highly frequent (63% to 66%) in both datasets. Similarly, for *TaSus1-7A,* haplotype Hap 1 was highly frequent in both datasets (62.9% to 71.9%), while haplotype Hap 2 was moderately frequent (21.9% to 32%).

**Table 1:** Frequencies in percent of reported favorable alleles in 14 candidate genes in two CIMMYT datasets.

Gene name	Chromosome	Favorable allele	Percent favorable allele	
			EYT2015-16	WAMI
*TaCwi-A1*	2A	*TaCwi-A1a*	0.6	6.4
*TaSus2*	2A	Hap-A	41.3	40.8
*TaSus2*	2B	Hap-H	0.4	0.3
*TaTGW6-A1*	3A	*TaTGW6-A1a*	57.0	86.0
*TaGS5*	3A	*TaGS5-A1a*	85.2	72.1
*TaCKX-D1*	3D	*TaCKX-D1a*	1.6	3.7
*TaCwi*	4A	Hap-4A-T	54.5	37.8
*TaCwi*	5D	Hap-5D-C	19.5	99.0
*TaTPP*	6A	TPP-6AL1a	96.5	87.7
*TaGW2*	6A	Hap-A/Hap-G	32.0/60.0	30.0/66.0
*TaGW2*	6B	Hap 1	27.4	29.2
*TaSus1*	7A	Hap 1/Hap 2	62.9/32.0	71.9/21.9
*TaGASR-A1*	7A	H1c	0.2	0.0

**Figure 1:**
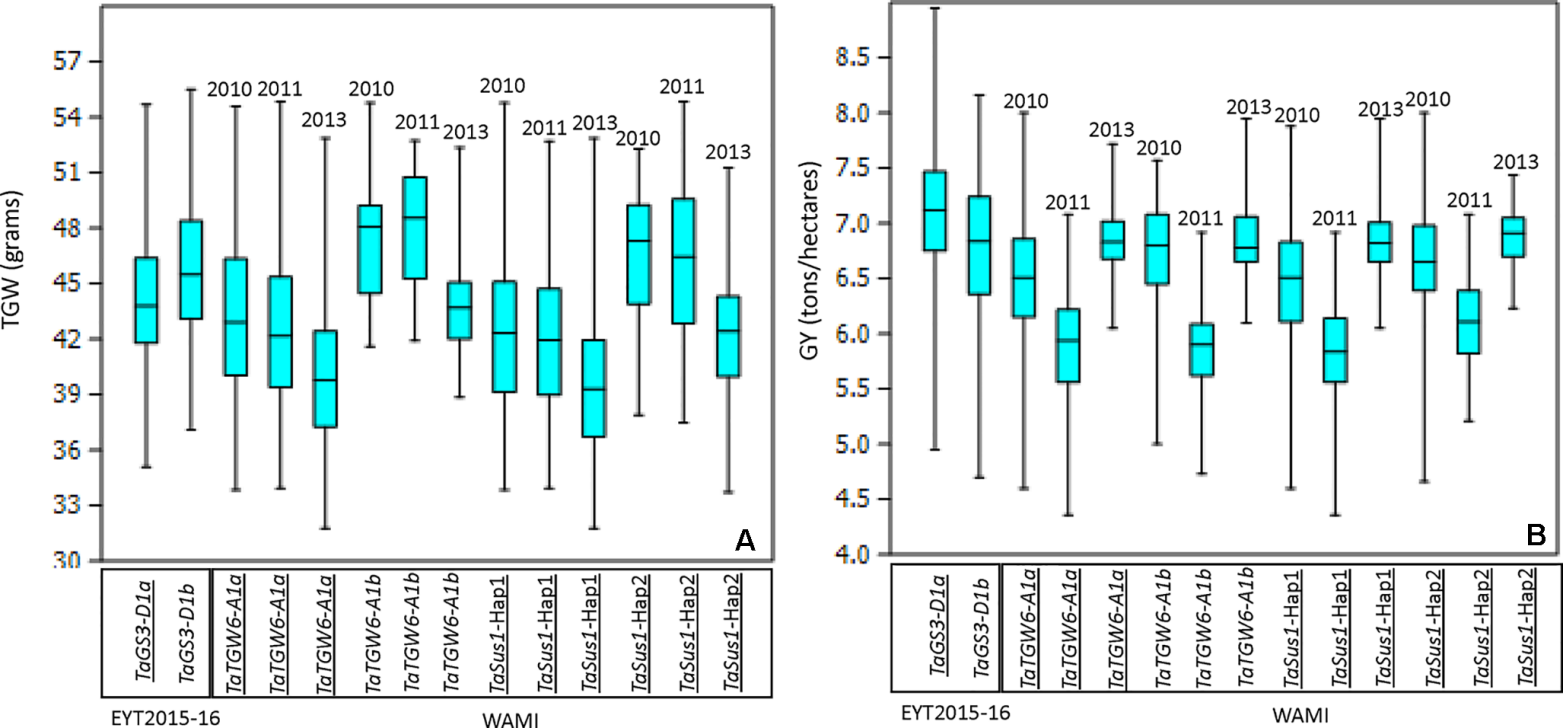
Allelic effects of two alleles of the associated genes *TaGS3-D1, TaTGW6-A1*, and *TaSus-1* in EYT2015-16 and WAMI, respectively, on TGW **(A)** and GY **(B)**. The reported favorable alleles in publications are underlined.

The CGs were initially mapped on chromosomes by blind association analysis, where the marker score was used as a phenotype (described in Lopes et al., 2015), in order to confirm the chromosome positions of the CGs. We obtained peaks at the respective chromosome except for *TaTGW6*. The gene was reported on chromosomes 3A and 4A in two different publications (Hanif et al., 2016; Hu et al., 2016). Blind association analysis mapped *TaTGW6* on chromosome 3A (**Figure S2**). In the EYT2015-16 dataset, only one gene (*TaGs3-D1*) showed significant association with TGW, while in WAMI, two genes (*TaTGW6* and *TaSus1*) showed associations (**Table 2**). The reported favorable alleles of the two genes, *TaGs3-D1a* (Zhang et al., 2014) and *TaTGW6_A1a* (Hanif et al., 2016), decreased TGW in our sets (**Figure 1A**). When their effects on GY were investigated, we found that in EYT2015-16, *TaGs3-D1a* increased GY, and in WAMI, *TaTGW6_A1a* decreased GY in 2010 and had no effect on GY in 2011 and 2013 (**Figure 1B**). Both alleles of *TaSus1-7A* are known to be favorable (Hou et al., 2014). In WAMI, Hap 2 was favorable increasing TGW and GY across the 3 years.

**Table 2:** Candidate gene association results in EYT2015-16 (EYT) and WAMI datasets.

Panel	Trait evaluation year	Candidate gene	*P* value	R^2^
EYT	2015–2016	*TaSus2-2A*	0.98779	1.55E-04
		*TaTGW6_3A*	0.05867	0.00899
		*TaGS5-3A*	0.10058	0.00733
		*TaCwi-4A*	0.78661	0.00128
		*TaCwi-5D*	0.77655	0.00133
		*TaGW2-6A*	0.86359	8.97E-04
		*TaGW2-6B*	0.00595	0.01459
		*TaSus1-7A*	0.21419	0.00527
		*TaTEF-7A*	0.69514	0.0017
		*TaGS3-D1*	3.49E-06	0.03345
WAMI	2010	*TaSus2-2A*	0.00202	0.05112
		*TaCwi-A1*	0.9838	1.16E-04
		*TaTGW6_3A*	1.51E-04	0.05322
		*TaGS5-3A*	0.363	0.01127
		*TaCKX-D1*	0.29451	0.01309
		*TaCwi-4A*	0.0694	0.02484
		*TaTPP-6AL*	0.08071	0.02365
		*TaGW2-6A*	0.08075	0.01769
		*TaGW2-6B*	0.39418	0.01054
		*TaSus1-7A*	5.67E-09	0.13644
		*TaTEF-7A*	0.15548	0.01842
		*TaGASR-A1*	0.60699	0.00353
		*TaGS3-D1*	0.70254	0.00501
WAMI	2011	*TaSus2-2A*	0.00621	0.04806
		*TaCwi-A1*	0.52438	0.00457
		*TaTGW6_3A*	1.30E-05	0.08617
		*TaGS5-3A*	0.06385	0.02549
		*TaCKX-D1*	0.31627	0.01248
		*TaCwi-4A*	0.24846	0.01454
		*TaTPP-6AL*	0.09631	0.02226
		*TaGW2-6A*	0.11556	0.01519
		*TaGW2-6B*	0.41188	0.01015
		*TaSus1-7A*	3.28E-08	0.12533
		*TaTEF-7A*	0.16706	0.01783
		*TaGASR-A1*	0.79193	0.00165
		*TaGS3-D1*	0.55303	0.00741
WAMI	2013	*TaSus2-2A*	0.00334	0.04734
		*TaTGW6_3A*	4.07E-05	0.07813
		*TaGS5-3A*	0.40908	0.01017
		*TaCKX-D1*	0.50073	0.00833
		*TaCwi-4A*	0.45735	0.00916
		*TaTPP-6AL*	0.94525	0.00133
		*TaGW2-6A*	0.49974	0.00489
		*TaGW2-6B*	0.60812	0.00646
		*TaSus1-7A*	5.15E-06	0.09204
		*TaTEF-7A*	0.11857	0.02053
		*TaGASR-A1*	0.7331	0.00219

### Haplotype Variation and Haplotype-Based GWAS for TGW in EYTs

Genome-wide, 501 haplotype blocks were constructed with a range from two to nine SNPs per block (**Supplementary Table S5**). The A, B, and D sub-genomes showed 197, 268, and 36 haplotype blocks and 506, 656, and 78 haplotypes, respectively.

**Figure 2:**
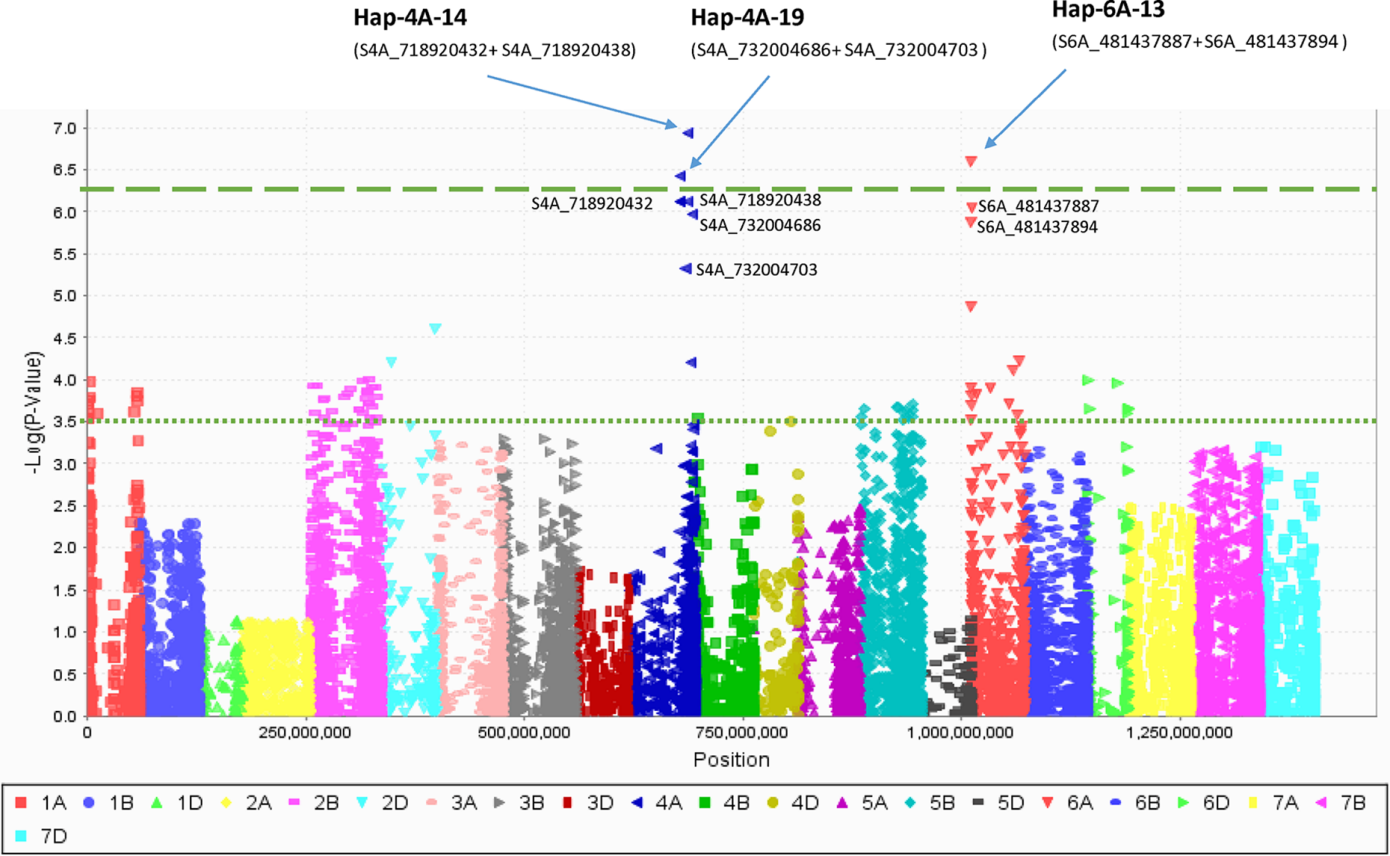
Manhattan plot created by combining haplotype blocks combined with single marker data. The haplotype blocks on 4A and 6A chromosomes had higher *P* value than their corresponding single SNPs (shown here) while haplotype block on 7A did not show any difference and not shown.

**Figure 3:**
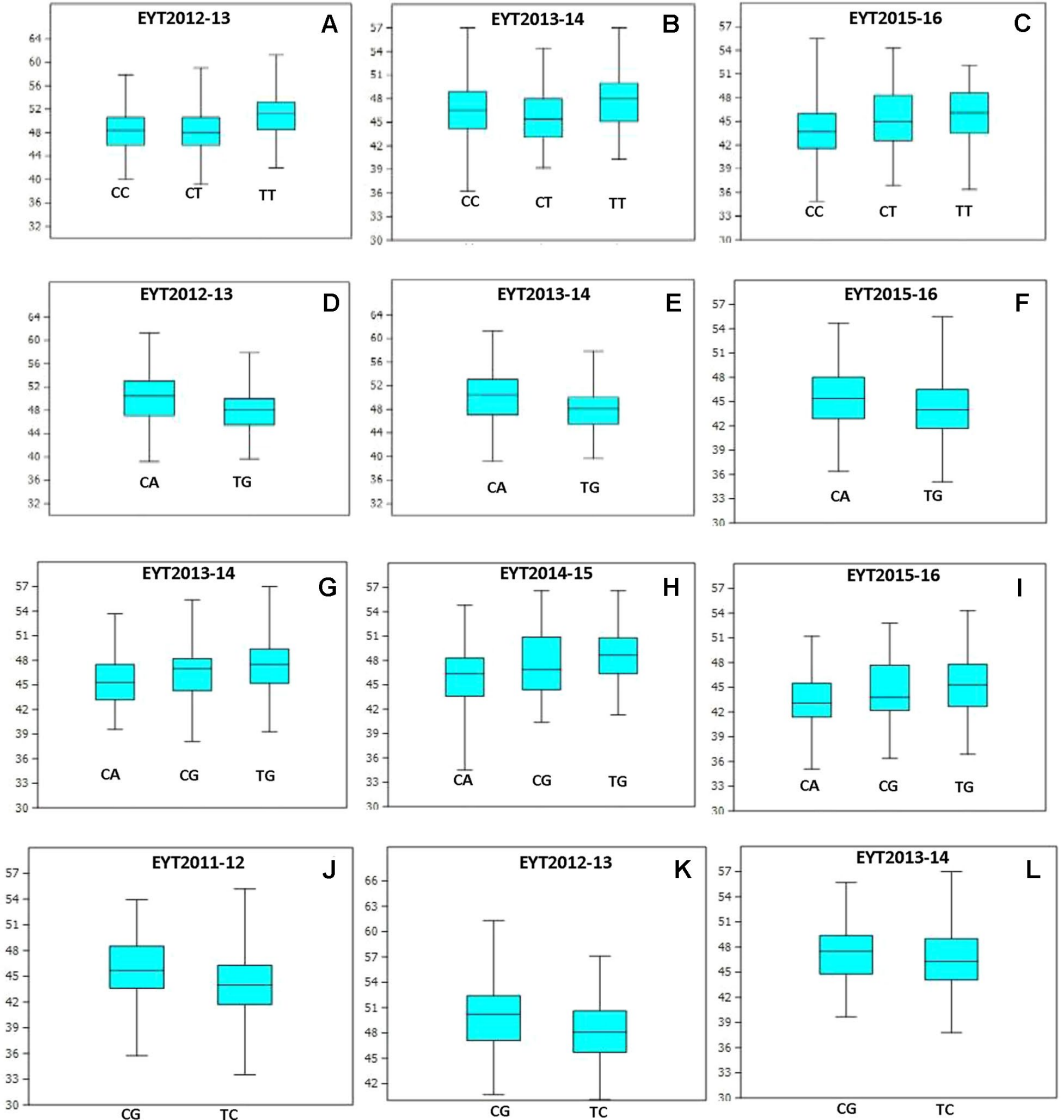
Allelic effects of haplotype in blocks Hap-4A-14 **(A–C)**, Hap-4A-19 **(D–F)**, Hap-6A-13 **(G–I)**, and Hap-7A-15 **(J–L)** on TGW across three EYTs.

**Figure 4:**
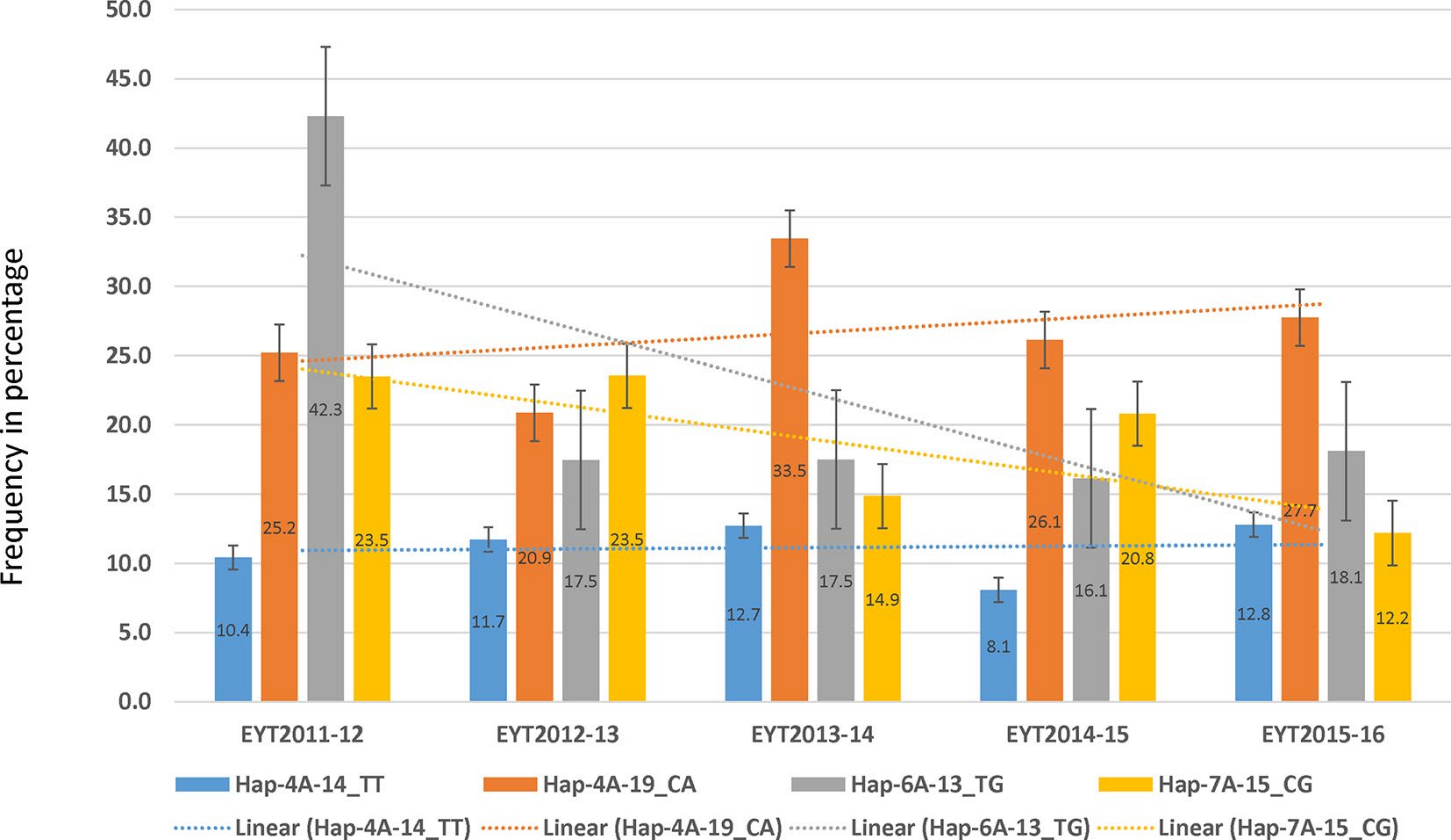
Allele frequencies (number shown in the center of the bars) of the four favorable haplotypes in five EYTs. The dotted line shows the trend observed in frequency of each of the favorable haplotype from one year to the other.

PCA in each of the five EYTs identified a moderate population structure in all panels: two to three groups (**Supplementary Figure S3**). Haplotype-based GWAS identified 15 haplotype blocks significantly associated with TGW. Four of them were associated with TGW in at least three EYTs and had large effects. These were classified as major associations (**Table 3**, **Figure 2** and **3**). Effects on GY of the four major TGW loci were investigated under multiple environments to investigate locus-specific tradeoffs between the two traits (**Table 3**). Two of the haplotype blocks were identified on chromosome 4A, Hap-4A-14 and Hap-4A-19. Hap-4A-14 had three haplotypes, CC, CT, and TT, of which TT was the favorable haplotype resulting in an increase of TGW by 1.39 to 2.74 g (**Table 3**). The GY advantage with this haplotype ranged from 0.9% to 6.7% across eight environments. The second block on chromosome 4A (Hap-4A-19) had two haplotypes: CA and TG. The favorable haplotype CA resulted in an increase of TGW by 1.22 to 2.40 g and GY advantage in five environments of 0.8% to 5.7%. The haplotype block identified on chromosome 6A (Hap-6A-13) showed three haplotypes, CA, CG, and TG, of which TG resulted in a 1.91 to 2.58 g increase in TGW and 1.4% to 9.4% yield advantage across seven environments. Hap-7A-15 on chromosome 7A revealed two haplotypes, CG and TC, of which CG was favorable with advantages in TGW and GY of 0.70 to 2.04 g and 0.5% to 2.4%, respectively. The frequencies of the favorable haplotypes at the four major TGW loci across EYTs are shown in **Figure 4**. The frequency of favorable haplotype Hap-4A-14-TT was almost stable across EYTs. Of the remaining three, Hap-4A-19-CA showed an increase while Hap-6A-13-TG and Hap-7A-15-CG showed a decreasing trend in frequency. Currently, we are designing KASP (Kompetitive Allele Specific PCR) assays for these four major loci for their validation.

**Table 3:** Four major TGW loci and their effects on GY across multiple environments and across EYTs. The favorable allele is shown in bold and is italicized.

Haplotype blocks	Mean TGW (g) with the corresponding haplotype			Mean GY (kg/ha) observed with the corresponding haplotype for TGW							
Hap-4A-14 S4A_718920432 S4A_718920438	EYT2012-13	EYT2013-14	EYT2015-16	EYT2012-13 SD	EYT2012-13F-5IR Irrigated	EYT2012-13 HS	EYT2013-14 B-5IR	EYT2013-14 SD	EYT2013-14 HS	EYT2015-16 B-5IR	EYT2015-16 B-2IR
CC	48.31	46.33	44.05	3,426	6,893	3,596	6,091	2,111	2,280	7,070	3,164
CT	48.37	45.46	45.28	3,401	6,884	3,563	6,188	2,092	2,195	7,085	3,228
***TT***	51.05	47.73	45.98	3,512	6,949	3,797	6,192	2,199	2,342	7,202	3,253
Percent increase in TGW and GY by the favorable allele	5.60%	3.00%	4.30%	3.20%	0.90%	6.50%	1.70%	5.10%	6.70%	1.90%	2.80%
Hap-4A-19 S4A_732004686 S4A_732004703	EYT2012-13	EYT2013-14	EYT2015-16	EYT2012-13 HS	EYT2013-14 F-5IR	EYT2015-16 F-5IR	EYT2015-16B-2IR	EYT2015-16 SD			
***CA***	50.40	47.39	45.50	3,737	6,124	7,136	3,227	1,663			
TG	48.00	45.89	44.29	3,568	6,022	7,074	3,170	1,573			
Percent increase in TGW and GY by the favorable allele	5%	3.20%	2.70%	4.70%	1.70%	0.80%	1.80%	5.70%			
Hap-6A-13 S6A_481437887 S6A_481437894	EYT2013-14	EYT2014-15	EYT2015-16	EYT2013-14 F-5IR	EYT2013-14 B-2IR	EYT2013-14 SD	EYT2014-15 SD	EYT2014-15 B-2IR	EYT2014-15 HS	EYT2015-16 SD	
CA	45.50	45.98	43.44	5,971	3,721	2,110	2,762	4,377	3,602	1,564	
CG	46.47	47.35	44.68	6,027	3,508	1,960	2,681	4,500	3,802	1,547	
***TG***	47.48	48.56	45.35	6,160	3,732	2,144	2,802	4,575	3,860	1,627	
Percent increase in TGW and GY by the favorable allele	4.30%	5.60%	4.30%	3.10%	6.40%	9.40%	4.50%	4.50%	7.10%	4.10%	
Hap-7A-15 S7A_80303639 S7A_80303659	EYT2011-12	EYT2012-13	EYT2013-14	EYT2011-12 B-5IR	EYT2011-12 SD	EYT2012-13 B-2IR	EYT2013-14 F-5IR	EYT2013-14 SD			
***CG***	45.85	50.23	47.17	8,068	2,713	4,776	6,152	2,132			
TC	44.01	48.19	46.47	7,895	2,649	4,701	6,068	2,085			
Percent increase in TGW and GY by the favorable allele	4.10%	4.20%	1.50%	2.20%	2.40%	1.60%	1.40%	2.2%			

Eleven minor TGW loci were identified on chromosomes 1B, 2A, 2B, 3A, 3B, 5D, 6A, and 6B (**Supplementary Table S6**). These were classified as minor because they were identified in only one or two EYTs and had small effects on TGW (less than 1.0 g increase in TGW). Squared correlation coefficient (*r^2^*), measure of LD, between the CGs and the associated haplotypes (**Table 4**) was very low varying from 0.004 to 0.183.

**Table 4:** Squared correlation coefficient between the identified TGW loci and candidate genes investigated in the present study.

Haplotype	Candidate gene(s)	Chromosome	*r*^2^
Major loci			
Hap-4A-14	*TaCwi*	4A	0.006
Hap-4A-19	*TaCwi*	4A	0.009
Hap-6A-13	*TaGW2*	6A	0.007
Hap-7A-15	*TaSus1*	7A	0.004
Minor loci			
Hap-2A-23	*TaSus2*	2A	0.034
Hap-3A-22	*TaGS5, TaTGW6*	3A	0.007, 0.013
Hap-6A-15	*TaGW2*	6A	0.183

### Epistatic Interactions Among TGW Loci and Stepwise Regression of Major Loci

Of the four major loci, three (Hap-4A-14, Hap-4A-19, and Hap-7A-15) were significantly involved in two or three locus interactions among themselves or with minor loci (**Supplementary Table S7**, **Figure S4**). The Hap-7A-15 was the main epistatic locus interacting with other identified loci contributing to additional variation of 4.4% to 11.6%.

**Figure 5:**
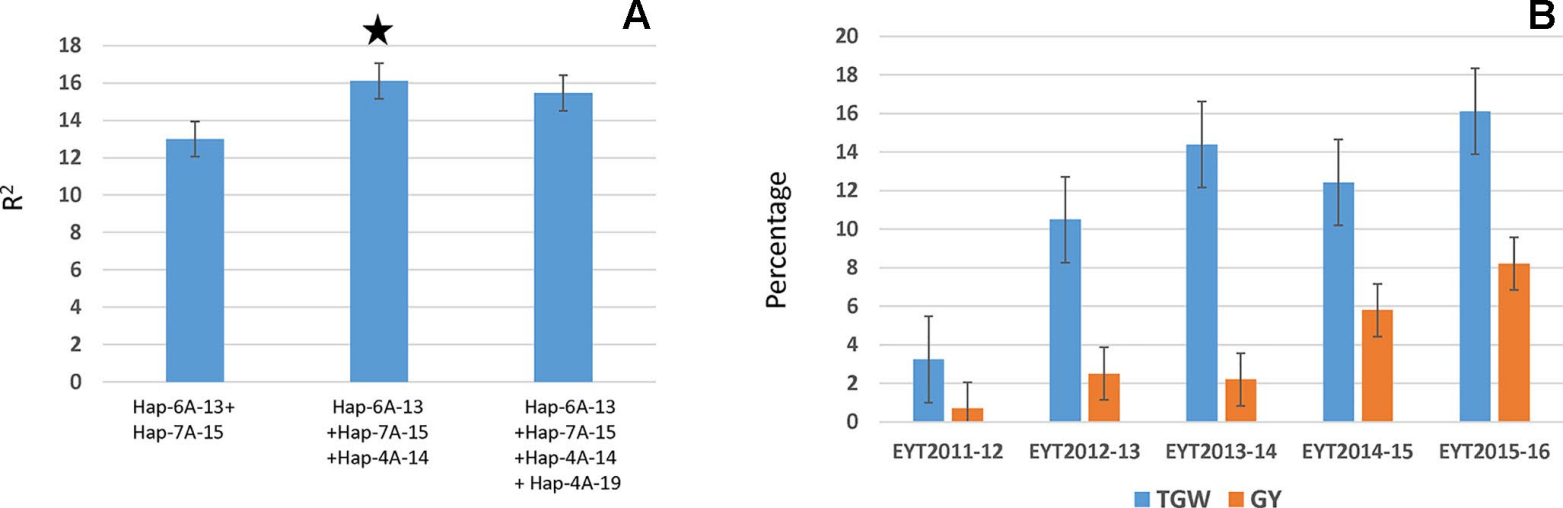
Stepwise regression of four major TGW loci **(A)** and percent increase in TGW and GY obtained in each EYT by combining the three best combination identified from stepwise regression **(B)**.

All four major loci were regressed on TGW in each individual EYT to identify the best combination, which would lead to increased TGW. A general pattern that emerged is summarized in **Figure 5A**. The highest percentage variation was contributed by a combination of the three haplotype blocks, Hap-6A-13, Hap-7A-15, and Hap-4A-14. Addition of the fourth locus, i.e., Hap-4A-19, decreased percentage variation. The three-locus combination resulted in a 3% to 16% and 0.7% to 8.2% increase in TGW and GY, respectively, in the EYTs (**Figure 5B**).

## Discussion

The allelic effects of CGs reported to increase TGW and/or grain morphometric traits have not been investigated comprehensively in diverse germplasm panels, thereby having limited impact in breeding. For instance, allele effects of CGs *TaGASR7-A*, *TaGW2*, *TaGS3-D1*, *TaCwi* (Dong et al., 2014; Qin et al., 2014; Zhang et al., 2014; Jiang et al., 2015), and recently *TaSnRK2.3* (Miao et al., 2017) have been examined exclusively in Chinese wheat. Zanke et al. (2015) and Mohler et al. (2016) tested the effects of 9 and 13 CGs on kernel traits in European winter wheat varieties. Both studies reported association of one or two genes with TGW in their datasets (*TaGW2-6A* in the former study and *TaSus-1-7A* and *TaCwi-A1* in the latter).

We investigated 14 CGs in two independent CIMMYT wheat datasets in order to validate their allele effects on TGW and to verify their suitability for MAS. We obtained moderate to high frequencies of the alleles/haplotypes for most genes except *TaSus2-2B, TaCKX6-D1*, and *TaGASR7-A1*. In congruence with Zanke et al. (2015) and Mohler et al. (2016), we obtained association of only one and two genes with TGW (*TaGs3-D1* in EYT2015-16 and *TaTGW6* and *TaSus1-7A* in WAMI). The reported favorable alleles of the two genes, *TaGs3-D1* and *TaTGW6* (Zhang et al., 2014; Hanif et al., 2016), decreased TGW in CIMMYT germplasm. Additionally, opposite and quite variable effects of the alleles on GY were observed. These results reinforce the previous findings that the currently known allelic effects of reported CGs related to TGW vary with genetic background and/or environment (Sukumaran et al., 2018), and it will be challenging to use the associated SNPs for MAS across individual breeding programs. Both favorable alleles were frequent in the two datasets, hence were not necessarily selected against by breeders. The opposing effect of *TaGs3-D1a* allele on TGW and GY in EYT2015-16 was similar to that reported by Mohler et al. (2016) for the *TaCwi-A1a* allele in a European winter wheat panel.

Some of CGs investigated have known function and have proven beneficial in Chinese wheat germplasm such as sucrose synthase 2 orthologous gene (*TaSus2*), putative cytokinin oxidase genes (*TaCKX2.1* and *TaCKX2.2*), cell wall invertase gene (*TaCwi*), etc. Cell wall invertase (CWI) is one of the key enzymes involved in establishing sink strength in various sink tissues. In maize, lack of expression of the endosperm-specific cell wall-invertase gene *Incw2* in the pedicel and endosperm caused interruption of photoassimilate transport into developing kernels, resulting in one-fifth of the normal seed weight (Miller and Chourey, 1992; Cheng et al., 1996; Kang et al., 2009). We investigated three wheat *CWI* genes reported on chromosomes 2A, 4A, and 5D, but none of them showed association with TGW in our panels. A similar case was observed with the sucrose synthase genes, playing an important role in the conversion of sucrose to starch, and the genes coding transcription and growth regulators, i.e., *TaTPP-6AL* (an important growth regulator involved in starch accumulation). On chromosome 6A, one of the first cloned grain width genes, *TaGW2*, showed effects with up to 3 g on TGW in Chinese wheat (Su et al., 2011). *TaGW2* is an orthologue of the *OsGW2* gene in rice that influences grain width and weight, and two haplotypes, Hap-6A-A and Hap-6A-G, have so far been reported (Su et al., 2011). Both haplotypes have been observed to be favorable (Su et al., 2011; Zhang et al., 2013). In the present study, although both alleles were present in moderate (Hap-6A-A) to high (Hap-6A-G) frequency, none showed association with TGW. The homeolog of *TaGW2* on 6B (*TaGW2-6B*) has been reported to have a stronger influence on TGW than *TaGW2-6A* (Qin et al., 2014), but none of the alleles of *TaGW2-6B* showed significant association with TGW in the present study. Thus, in the CIMMYT wheat breeding program it will be difficult to apply the investigated gene-based markers in MAS unless re-tested in specific populations to identify beneficial genetic backgrounds. The reported favorable alleles have mainly been identified from SNP(s) or INDELs observed in promotors or intron sequences of genes in a narrow set of Chinese wheat lines. For example, published haplotypes (Hap-6A-A and Hap-6A-G)-based markers for the grain width gene *TaGW2*-6A are based on 593A/G SNP out of 8 SNPs identified in 2.9 kb sequenced promotor region in 34 Chinese wheat accessions (Su et al., 2011). Zanke et al. (2015) reported a novel allele for the *TaGs3-D1* gene, named as *TaGs3-D1c*, in European winter wheat, which was absent in Chinese wheat. The existence of additional alleles yet unobserved for the CGs, together with significant genotype × genotype and/or genotype × environment effects, might be likely explanation for the varying results among studies. Allele mining of promotors and intron regions of the CGs in diverse set of lines should be a priority research area to identify novel alleles associated with TGW.

In order to explore the genetic determinants of TGW in CIMMYT germplasm, we adopted a haplotype-based GWAS approach (N’ Diaye et al., 2017; Singh et al., 2018) and identified four stable loci across EYTs. On chromosome 4A, the two identified loci, Hap-4A-14 and Hap-4A-19, were neither in LD (*r^2^* = 0.02) with each other nor with the gene *TaCwi-4A*. The two loci identified on chromosome 4A are therefore independent and likely novel. The major locus on chromosome 6A (Hap-6A-13) had the largest effect on both TGW and GY with increases of up to 2.60 g and 258 kg/ha in TGW and GY, respectively. Hap-6A-13 was not in LD with *TaGW2-6A* and is located on 6AL based on BLAST analysis (https://plants.ensembl.org/Triticum_aestivum/Info/Index), while *TaGW2-6A* is mapped on 6AS near the centromere (Su et al., 2011). Sukumaran et al. (2018) identified a locus on 6A in the WAMI panel having positive association for GY and TGW with allelic substitution effects of 1.7% and 4% on TGW and GY, respectively. In the present study, the favorable allele at the 6A locus (“TG”) contributed to allelic effects of up to 5.6% and 9.4% on TGW and GY, respectively. The LD between 6A locus in WAMI and Hap-6A-13, on an average, is 0.05, suggesting these two as different loci.

Three TGW genes (*TaAGP-S1*, *TaTGW*, and *TaSus-1*) are known on chromosome 7A in wheat (Hou et al., 2014; Hu et al., 2016; Hou et al., 2017). Of these, *TaSus-1* was investigated in the current research and it showed association with TGW in the WAMI panel. The *r^2^* values between Hap-7A-15 and *TaSus1-7A* was 0.004, which eliminates the possibility of Hap-7A-15 being *TaSus1-7A*. BLAST analysis showed that *TaTGW* and *TaAGP-S1* are ∼125 and ∼262 Mb upstream of Hap-7A-15, respectively, which also precludes the possibility of Hap-7A-15 as one of these genes. Hap-7A-15 was in fact located in the middle of two genes within 1 Mb region; TraesCS7A02G124500 is ∼0.9 Mb downstream and TraesCS7A02G124600 is ∼0.1 Mb upstream. TraesCS7A02G124500 is implicated in transferase activity (transferring acyl groups other than amino-acyl groups) and TraesCS7A02G124600 in serine-type endopeptidase activity. In model crop rice, role of serine-type peptidase encoding genes in determining grain size and grain filling has been demonstrated (Li et al., 2011; Li et al., 2016). For example, *OsGS5* (encoding a putative serine carboxypeptidase) was identified to promote cell division by regulating cell cycle genes resulting in large grain size by an increased cell number (Li et al., 2011). Hence, we speculate that Hap-7A-15 could be linked to such cell cycle-related genes.

TGW and grain number (GN) are the two primary determinants of GY in wheat. While the association between GY and GN is generally positive, the positive association of GY with TGW is less profound and sometimes even negative as was also observed in the present study (**Supplementary Table S4**). Hence, identification of QTL that increases TGW without decreasing GY or alternatively QTL displaying a low trade-off between the two traits could be beneficial for improving wheat GY. Many previous studies have reported stable QTLs for TGW on chromosomes 2B, 2D, 4B, 6A, and 7D using bi-parental populations (Simmonds et al., 2014; Lopes et al., 2013; Yan et al., 2017; Tahmasebi et al., 2017) and on 2B, 4A, 4B, and 6A using GWAS (Guan et al., 2018; Sukumaran et al., 2018; Mangini et al., 2018). However, compensatory effect of TGW enhancing QTL on GY has not been addressed much (Zhai et al., 2018; Guan et al., 2018; Sukumaran et al., 2018). The positive allele effects of all four identified loci on TGW and GY in the current research brings in opportunities to generate new combination of TGW alleles, which might increase overall GY when introgressed in lines with high GN. It is noteworthy that contributions of these loci to GY became even more important at higher levels of stress (e.g., mild drought, severe drought, or heat stress) (**Table 3**). Forward stepwise regression identified a superior combination of three haplotype blocks conferring high TGW and GY in EYTs (3% to 16% and 0.7% to 8.2%, respectively).

The significance of epistasis in the genetic architecture of TGW has been investigated in wheat using bi-parental populations (Wu et al., 2012; Lv et al., 2014; Wei et al., 2014). Using GWAS, such studies are rare (Sehgal et al., 2016). Unravelling QTL with epistatic effects using GWAS will have more potential compared to linkage mapping because fixation of QTL alleles will be less likely in a naturally segregating population, and the power of GWAS will be considerably larger (Sehgal et al., 2016, Sehgal et al., 2017). This was reflected in the current study, where the cumulative contribution from epistatic effects was higher than that from main effects (**Table S4**). The locus Hap-7A-15 on chromosome 7A interacted with other major and minor loci on chromosomes 3A, 3B, 4A, 6A, and 6B in two- and three-locus interactions contributing to up to 8.9% and 11.6% variation, respectively. Bi-parental designs, on the other hand, revealed variation of 1.8% to 3.8% (Lv et al., 2014; Wei et al., 2014).

## Conclusions

The findings of the study provide a useful foundation for more detailed investigations on genetic background dependence and environment sensitivity of the known CGs for TGW. The current available gene-based markers are hardly deployable in CIMMYT wheat germplasm, unless re-tested in specific genetic backgrounds. The direct use of gene-based markers reported in the literature is therefore in general questionable especially for more complex trait. More and better marker validation is required, which is currently lacking and has not received adequate attention. We have identified four stable and novel TGW loci in CIMMYT wheat germplasm. The positive allelic effects of the identified loci on GY and TGW make them attractive targets for MAS at CIMMYT.

## Data Availability Statement

All datasets for this study are included in the manuscript and the **Supplementary Files**.

## Author Contributions

DS and SD conceived the manuscript. SD and CF generated candidate genes-based marker data. DS analyzed the data and wrote the manuscript. SM, RS, and CG generated phenotypic data. GGB validated KASP assays designed for major loci. All authors reviewed the manuscript.

## Funding

The work was supported by funding from CRP WHEAT and the Delivering Genetic Gains in Wheat (DGGW) project (OPPGD1389) funded by the Bill & Melinda Gates Foundation (BMGF), the UK Department for International Development (DFID), and the US Agency for International Development (USAID) Feed the Future Innovation Lab for Applied Wheat Genomics (Cooperative Agreement No. AID-OAA-A-13-00051).

## Conflict of Interest

The authors declare that the research was conducted in the absence of any commercial or financial relationships that could be construed as a potential conflict of interest.
