# Supplementary material for: Validation of Candidate Gene-Based Markers and Identification of Novel Loci for Thousand-Grain Weight in Spring Bread Wheat
Source: Front Plant Sci. 2019 Sep 26;10:1189. doi: 10.3389/fpls.2019.01189 (PMC6775465; doi:10.3389/fpls.2019.01189)
Supplement: Supplementary file 8 [file DataSheet_1.pdf]

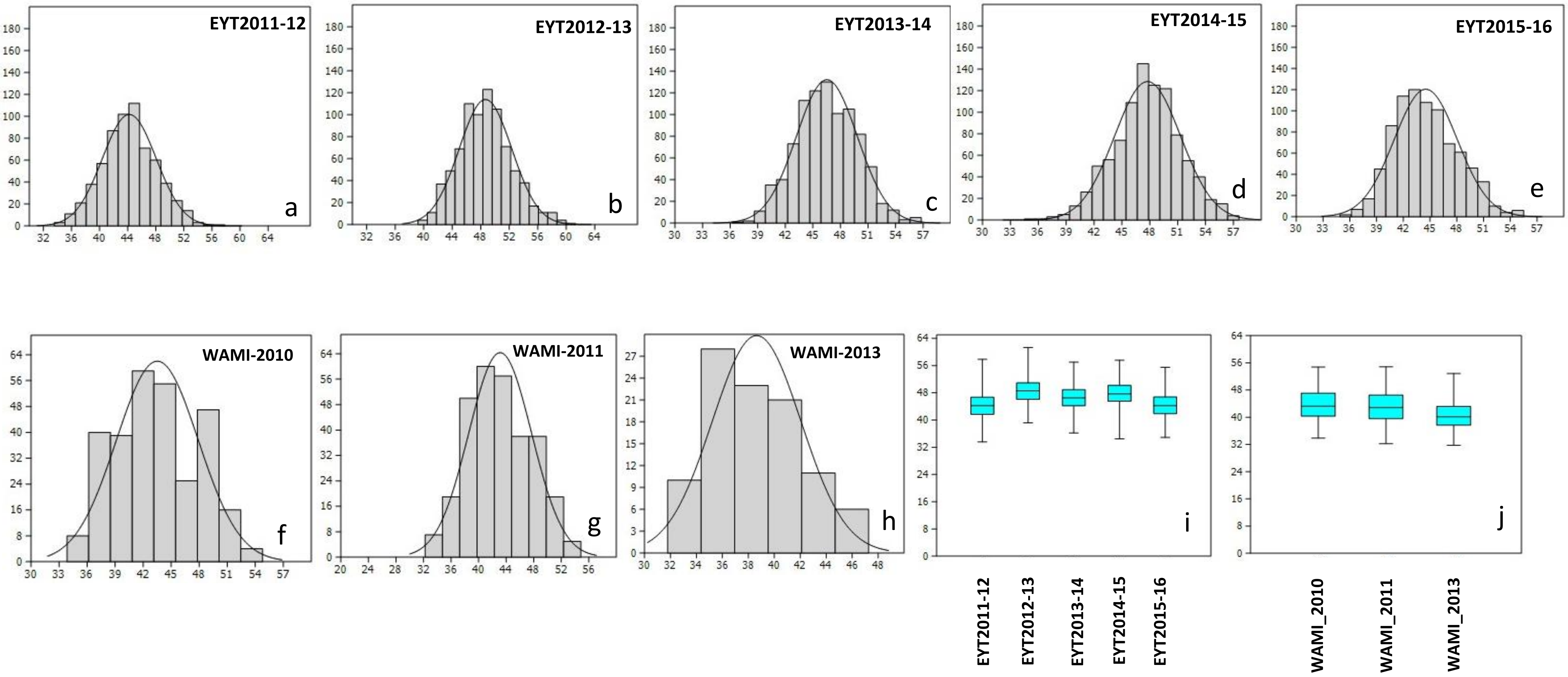

Fig. S1 Distribution of TGW in five EYTs (a-e) and in WAMI (f-h). Box plots showing means of TGW in EYTs (g) and WAMI (h).
