# Supplementary material for: Validation of Candidate Gene-Based Markers and Identification of Novel Loci for Thousand-Grain Weight in Spring Bread Wheat
Source: Front Plant Sci. 2019 Sep 26;10:1189. doi: 10.3389/fpls.2019.01189 (PMC6775465; doi:10.3389/fpls.2019.01189)
Supplement: Supplementary file 9 [file DataSheet_2.pdf]

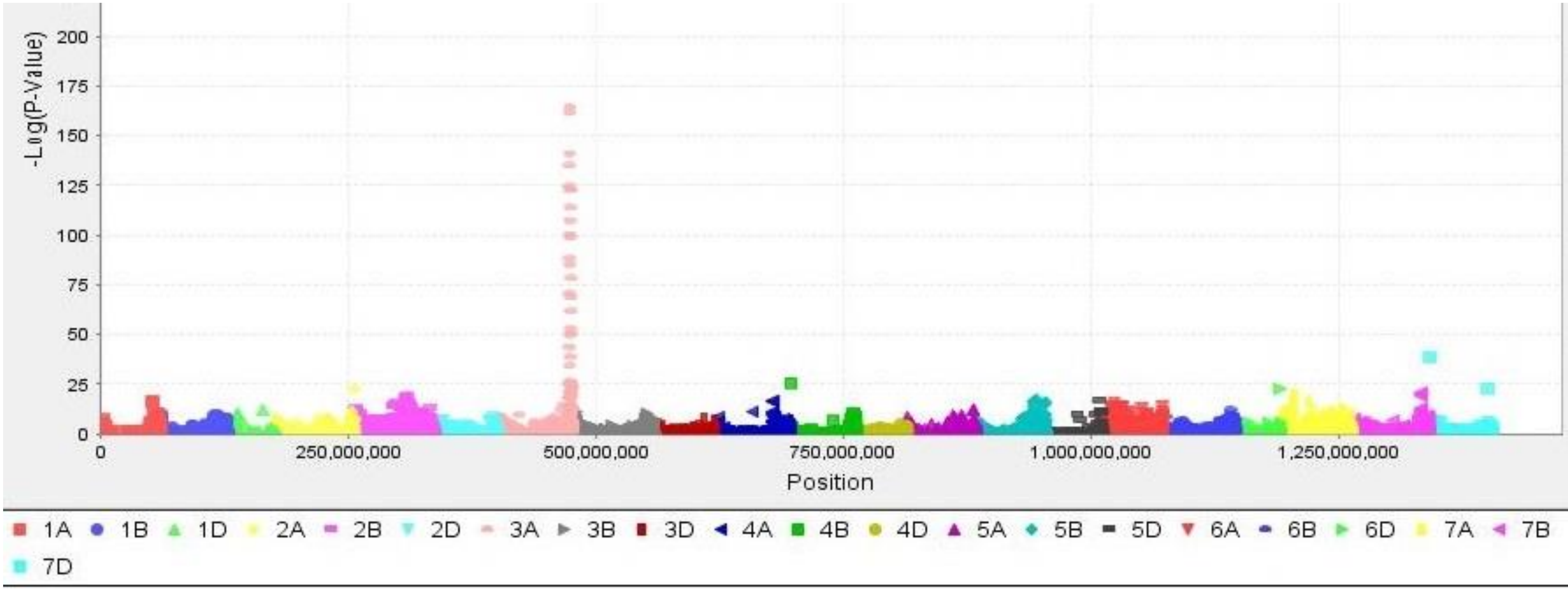


Fig. S2 Manhattan plots showing mapping of *TaTGW6* gene on chromosome 3A using blind association analysis
