# Supplementary material for: Validation of Candidate Gene-Based Markers and Identification of Novel Loci for Thousand-Grain Weight in Spring Bread Wheat
Source: Front Plant Sci. 2019 Sep 26;10:1189. doi: 10.3389/fpls.2019.01189 (PMC6775465; doi:10.3389/fpls.2019.01189)
Supplement: Supplementary file 10 [file DataSheet_3.pdf]

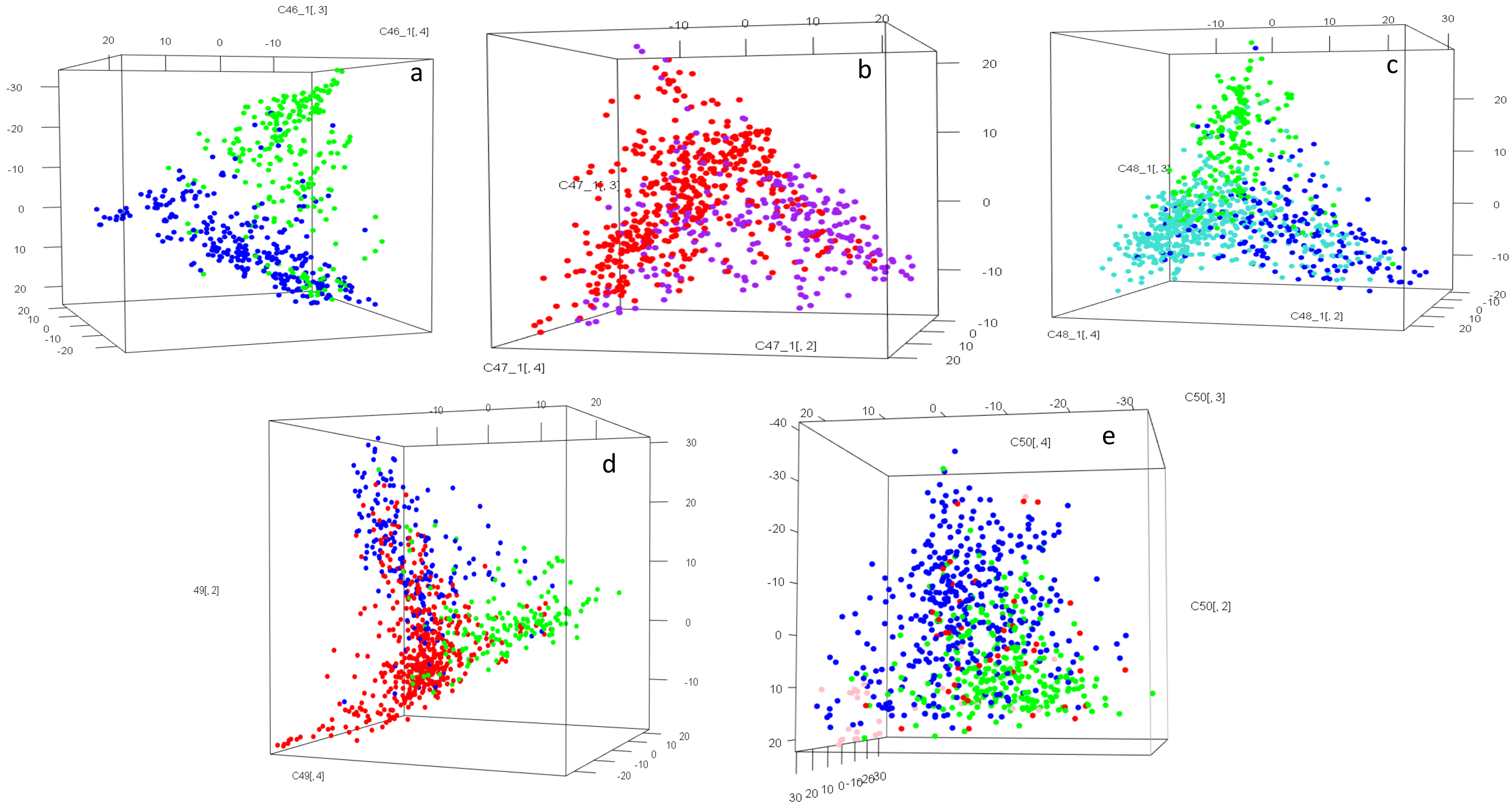

Fig. S3 Three dimensional principal component analysis plots in five EYTs; EYT2011-12 (a), EYT2012-13 (b), EYT2013-14 (c), EYT2014-15 (d) and EYT2015-16 (e).
