# Supplementary material for: Validation of Candidate Gene-Based Markers and Identification of Novel Loci for Thousand-Grain Weight in Spring Bread Wheat
Source: Front Plant Sci. 2019 Sep 26;10:1189. doi: 10.3389/fpls.2019.01189 (PMC6775465; doi:10.3389/fpls.2019.01189)
Supplement: Supplementary file 11 [file DataSheet_4.pdf]

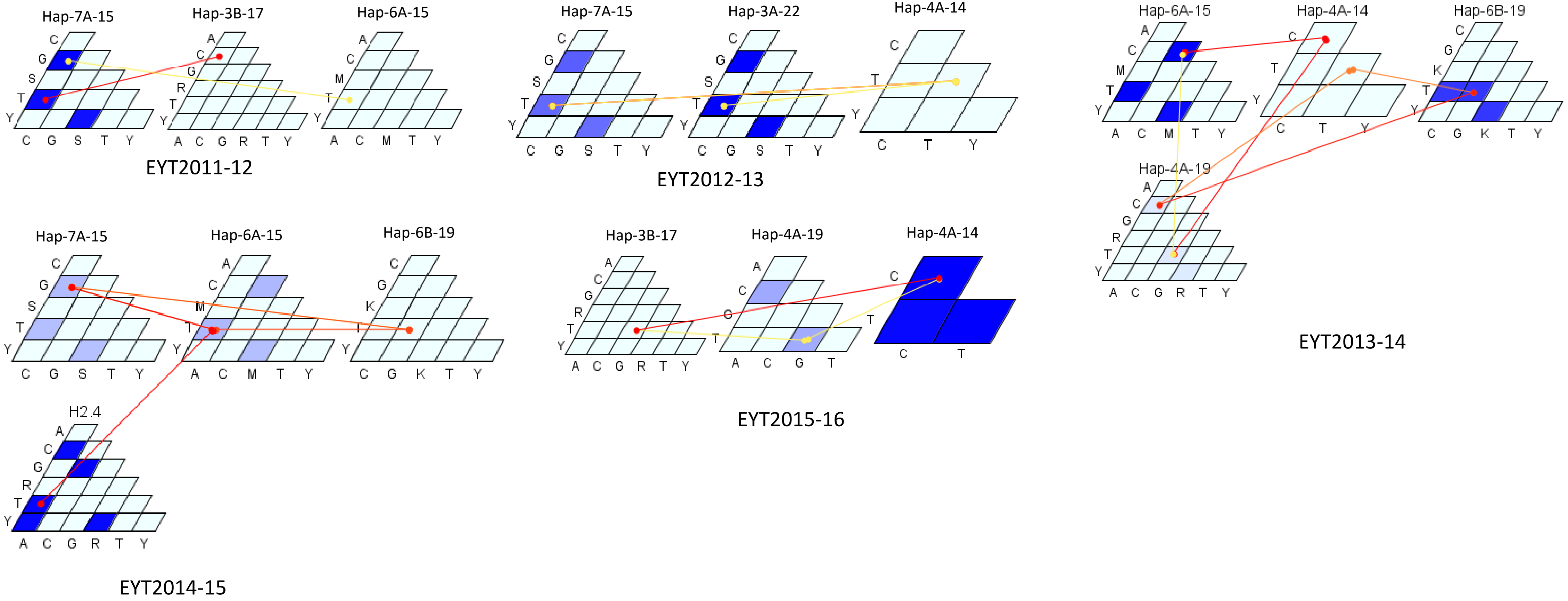

Fig. S4 Epistatic interactions among major and minor effect loci for TGW. The magnitude of marker effect (F value) is represented with shades of blue (dark blue with stronger interaction). The magnitude of epistatic interaction is presented with colors from yellow to red (stronger interaction)
